# Supplementary material for: Three-dimensional spin-wave dynamics, localization and interference in a synthetic antiferromagnet
Source: Nat Commun. 2024 Apr 9;15:3057. doi: 10.1038/s41467-024-47339-9 (PMC11004151; doi:10.1038/s41467-024-47339-9)
Supplement: Supplementary file 1 — Supplementary Information [file 41467_2024_47339_MOESM1_ESM.pdf]

Supplementary Information for

Three-dimensional spin-wave dynamics, localization and interference in a synthetic antiferromagnet

Davide Girardi[1], Simone Finizio[2], Claire Donnelly[3,4], Guglielmo Rubini[1], Sina Mayr[2,5], Valerio Levati[1], Simone Cuccurullo[1], Federico Maspero[1], Jörg Raabe[2], Daniela Petti[1]*, Edoardo Albisetti[1]*

[1]Dipartimento di Fisica, Politecnico di Milano; Piazza Leonardo da Vinci 32, Milano 20133, Italy.

[2]Swiss Light Source, Paul Scherrer Institut; Forschungsstrasse 111 5232 PSI Villigen, Switzerland.

[3]Max Planck Institute for Chemical Physics of Solids; Nöthnitzer Str. 40, 01187 Dresden, Germany.

[4]International Institute for Sustainability with Knotted Chiral Meta Matter (WPI-SKCM2), Hiroshima University; Hiroshima 739-8526, Japan.

[5]Laboratory for Mesoscopic Systems, Department of Materials, ETH Zurich; 8093 Zurich, Switzerland.

*Corresponding author. Email: daniela.petti@polimi.it (DP); edoardo.albisetti@polimi.it (EA)

1. Magnetic characterization of the SAF film

Supplementary Figure 1 shows the hysteresis loop of the CoFeB (50) / Ru (0.5) / NiFe (40) synthetic antiferromagnetic (SAF) film acquired by a vibrating sample magnetometer (VSM, Microsense EZ9) at room temperature. The VSM loop shows the in-plane component of the magnetization in the as grown sample, normalized to its maximum, as a function of the in-plane external magnetic field in the range -50 mT, +50 mT. The orange and teal arrows show the direction of the magnetization in the NiFe and CoFeB layers, respectively. For strong negative (positive) field, the magnetization of the two layers is saturated in the negative (positive) direction. As the field decreases, the RKKY-mediated coupling forces the orientation of the magnetizations towards an antiparallel alignment. The difference in the saturation magnetizations of the two layers gives rise to a non-compensation of the magnetic moments in the antiparallel configuration, so that a small hysteresis loop is visible at low fields.

2. Reconstruction of the static magnetization

Supplementary Figure 2 shows the static magnetization of the NiFe (panel A) and CoFeB (panel B) layers at remanence, with the arrows indicating point-by-point the magnetization, while the color-code represents the in-plane magnetization direction with respect to the *x* axis. A curl of the magnetization in the clockwise (counter-clockwise) direction for the NiFe (CoFeB) is visible and arises from the shape anisotropy of the microstructure. Point-by-point the coupling of the two magnetizations of the two films is antiparallel, as expected in the SAF structure. Deviations from the ideal antiferromagnetic coupling are observed close to the edges, likely due to variations in the

interlayer coupling induced by the non-uniform thicknesses at the edges. The spin textures are indicated by a black point and dashed lines: a vortex and two sharp domain walls are stabilized.

3. Spin-wave dispersion of the acoustic mode in SAF

Supplementary Figure 3 reports the micromagnetic simulation of the dispersion of the acoustic mode in our SAF sample. In the simulation, spin waves are excited by an out-of-plane sinc-shaped field pulse $b(t) = b_0 \frac{\sin(2\pi f_0(t-t_0))}{2\pi f_0(t-t_0)}$, with amplitude b_0 = 10 mT and frequency f_0 = 30 GHz. The field was applied in a narrow line at the center of the simulated area. The total simulated volume was 20.48 µm x 20 nm x 90 nm, discretized into cells having dimensions of 5 × 5 × 5 nm^3. The dispersion relation was then extracted by calculating the Fourier-transform in space and time of the *z*-component of the magnetization in the whole simulated area.

The strong non-reciprocity of the SAF is confirmed by observing the $+k$ (red) and $-k$ (blue) branches[1,2]. The green point represents our experimental data point (f = 0.86 GHz, k = 0.97±0.07 10^7 rad/m); it is worth to notice that at the excitation frequency of our experiment, the propagation of the mode is unidirectional (see Supplementary Figure 3b), resulting in the absence of back-reflection from the boundaries or defects.

4. Definition of $\Delta\theta$ and sign rule

The in-plane dynamic angle $\Delta\theta$ is defined as the angle formed by the in-plane component of the dynamic magnetization associated with the precession of the spins and the average direction of the magnetization over one period of oscillation (see main text and Supplementary Figure 4). Due to the antiparallel coupling of the in-plane component of the SAF spin-wave modes, the in-plane component of the dynamic magnetization is always antiparallel in the two layers. To better visualize this, the sign convention for $\Delta\theta$ is opposite in the two layers, as shown in Supplementary Figure 4.

5. Simulations of the *z*-dependence of the spin-wave amplitude in a compensated SAF

To better understand the underlying physical phenomena giving rise to the *z*-dependent variation of the in-plane and out-of-plane SW amplitudes, additional simulations with a multilayer based on two antiferromagnetically coupled CoFeB layers of 45 nm each was chosen. Supplementary Figure 6a shows a sketch of the simulated structure. The material parameters used in the simulation were the following: M_s CoFeB = 1250 kA m^{-1}, exchange constant A_{ex} CoFeB = 0.75 × 10^{-11} J m^{-1}, and interlayer exchange coupling constants J = -1.2 mJ m^{-2}. The Gilbert damping was set to α CoFeB = 0.008. The total simulated volume was discretized into cells having dimensions of 5 × 5 × 5 nm^3.

SWs were excited by a sinusoidal out-of-plane magnetic field at 0.86 GHz localized in narrow region at the center of the simulated volume.

In Supplementary Figure 6 we plot the variations of the in-plane $A_{\Delta\theta}$ (Supplementary Fig. 6b) and out-of-plane $A_{\Delta\varphi}$ (Supplementary Fig. 6c) amplitudes with respect to their values at the bottom CoFeB surface. In this case, the in-plane SW amplitude is almost constant through the whole thickness of the multilayer, while the out-of-plane SW amplitude is maximum in the middle of the structure and decreases symmetrically towards the top and bottom surfaces. In this case, as the wavelength of the spin waves and penetration depth of the Damon-Eshbach modes is longer than the thickness of the films, the in-plane amplitude is expected to be uniform through the thickness, as already predicted in Ref [3].

6. Spatial and temporal evolution of the saddle-shaped 3D interference figure

To understand the spatial and temporal evolution of the *z*-dependent saddle-shaped destructive interference structure we further analyze the micromagnetic simulations. In Supplementary Figure 10-11 we show the spatial dependence of the three-dimensional interference. Panel a shows the top-view of a snapshot of the simulated time-resolved magnetization within the destructive interference region. The interference arises from the superimposition of two spin waves beams in antiphase and propagating at an angle of ~ 50° with respect to each other. In panel b of Supplementary Figure 10 we show several vertical sections, extracted in correspondence of the numbered black lines in panel a. The white regions correspond to zones where the in-plane dynamic angle $\Delta\theta = 0°$, i.e. where the in-plane component of the spin wave changes sign, therefore separating the vertical section into three "lobes" indicated as "a", "b" and "c". Far away from the interference region (Vertical section 1), the $\Delta\theta = 0°$ regions appear as two vertical lines separating the lobes. As we approach the interference region (Vertical sections 2, 3), the central lobe "b" progressively shrinks in size and magnitude, as an effect of the superimposition of the two waves. As a result, the two $\Delta\theta = 0°$ regions gradually tilt and eventually merge within the bottom CoFeB layer (Vertical sections 4, 5), giving rise to the saddle-shaped $\Delta\theta = 0°$ structure we observe.
Both the spatial and temporal dependence of the three-dimensional destructive interference structure originate from the superposition of spin waves with *z*-dependent in-plane SW amplitude (see Figure 3 and related discussion in the main text), which presents its minimum at the bottom CoFeB surface.

To gain a comprehensive view of the phenomenon, we developed a simple general model for calculating and visualizing in three dimensions the interference pattern generated by two planar sinusoidal waves. Let us assume a reference system in which *x*,*y* are the horizontal axes (corresponding in our experiment to the CoFeB film plane) and *z* is the vertical axis (in our experiments the CoFeB film depth).
Consistent with the *z*-dependence of the amplitude of our experiments (see Fig. 3), we implemented in our MATLAB code the possibility to have a linear dependence of the amplitudes of the waves $A_1(z)$ and $A_2(z)$ along the depth *z*, expressed by:

$$A_1(z) = A_{1b} + \frac{A_{1t} - A_{1b}}{h} z, \qquad A_2(z) = A_{2b} + \frac{A_{2t} - A_{2b}}{h} z,$$

where A_{1b} (A_{2b}), A_{1t} (A_{2t}) are the amplitudes at the surface at *z* = 0 and *z* = *h*, with *h* the total film thickness.
The full spatio-temporal profile of the two waves is therefore described by:

$$\theta_1(t, \mathbf{r}) = A_1(z)\cos(k_{x1}x + k_{y1}y - \omega_1 t), \;\; \theta_2(t, \boldsymbol{r}) = A_1(z)\cos(k_{x2}x + k_{y2}y - \omega_2 t)$$

Where k_x and k_y are the *x* and *y* components of the wavevectors, respectively, propagating at angles α_1 and α_2 with respect to the horizontal axis, and defined as follows:

$$k_{x1} = 2\pi/\lambda_1 \cos(\alpha_1), k_{y1} = 2\pi/\lambda_1 \sin(\alpha_1), k_{x2} = 2\pi/\lambda_2 \cos(\alpha_2), k_{y2} = 2\pi/\lambda_2 \sin(\alpha_2)$$

The frequency of the waves are:

$$\omega_1 = 2\pi f_1, \quad \omega_2 = 2\pi f_2.$$

The three-dimensional interference pattern, resulting from the superposition of the two waves is therefore:

$$\theta_{\text{int}}(t,\mathbf{r}) = \theta_1(t,\mathbf{r}) + \theta_2(t,\mathbf{r})$$

Where **r** is the position vector. From this expression, it is possible to directly extract the amplitude of the oscillation $A_{\theta_{\text{int}}}(\mathbf{r})$ point-by-point in the three-dimensional space, via numerical Fast-Fourier-Transform. Noteworthy, in our case the wave frequencies $f_1 = f_2$, since they are fixed by the excitation field frequency, therefore $A_{\theta_{\text{int}}}(\mathbf{r})$ is constant in time. On the opposite, superposition of waves with different frequencies would give rise to time-dependent amplitudes and "beating" effects.

For reproducing the experimental conditions observed in the reconstruction of spin-wave dynamics in the CoFeB layer, we used a wavelength $\lambda_1 = \lambda_2 = 650$ nm, which is equal to the experimentally measured spin-wave wavelength, and a frequency $f_1 = f_2 = 0.86$ GHz, equal to the excitation field frequency. The thickness of the CoFeB film is $h = 40$ nm. The angles of the wavevectors were set to $\alpha_1 = 180°$ and $\alpha_2 = 230°$, giving rise to experimental conditions similar to Fig. 4a, where the two wavefronts are propagating from right to left at an angle of 50° with respect to each other. Regarding the wave amplitudes, we considered two *z*-dependent amplitude profiles $A_1(z)$ and $A_2(z)$, both decreasing linearly from the top to the bottom surface, with a slightly different decrease rate as a function of *z*, consistently with our findings of Fig. 3. This can originate from the different propagation angle and therefore dispersion of the two waves, their different excitation efficiency, or their interaction with the locally non-uniform texture. In this scenario, Supplementary Fig. 12 **a, b** shows top-view (*xy* plane) snapshot at *t* = 0 of the two individual waves.

Now let us consider the interference $\theta_{\text{int}}(t,\mathbf{r}) = \theta_1(t,\mathbf{r}) + \theta_2(t,\mathbf{r})$ generated by the spatial superposition of these two waves. Supplementary Fig. 12 **c** shows a snapshot at $t = 0$ s of the interference pattern. Since the wavefronts propagate at an angle, the resulting interference pattern features spatially alternating interference minima and maxima arising from regions with destructive and constructive interference, respectively. The direction of propagation of the interfering wavefronts is indicated as black arrows. We note that this interference figure well reproduces the experimental one shown in Fig. 4a and the one obtained via micromagnetic simulations shown in Fig. 4b. The geometry of the interference pattern is particularly clear from the corresponding "amplitude" image, which shows point-by-point the amplitude $A_{\theta_{\text{int}}}(\boldsymbol{r})$ of the oscillation. In particular, the central dark region marks a region where the two waves superimpose in antiphase giving rise to destructive interference and therefore 0 amplitude.

We now analyze the interference along the vertical direction *z*, in panels Supplementary Fig. 12 **e**, **f**. In panel **e**, the $\theta_{\text{int}} = 0$ surfaces are visualized in white, and feature the characteristic buried "saddle" shape we observe experimentally. It is clear that the buried saddle points (e.g. the one in green) are located where the destructive interference region intersects the perpendicular propagating wavefronts. The three-dimensional geometry of the destructive interference region is clear in the corresponding $A_{\theta_{\text{int}}}(\mathbf{r})$ image of panel **f**. Here, it is possible to appreciate the fact that the destructive interference region, where $A_{\theta_{\text{int}}} = 0$, is buried in the middle of the layer (black "tube" in panel **f**), and is composed by the points occupied by the "saddle" at different times during propagation.

References

1. Wintz, S. *et al.* Magnetic vortex cores as tunable spin-wave emitters. *Nature Nanotech* **11**, 948–953 (2016).

2. Albisetti, E. *et al*. Optically Inspired Nanomagnonics with Nonreciprocal Spin Waves in Synthetic Antiferromagnets. *Adv. Mater*. **32**, 1906439 (2020).
3. Grünberg, P. Magnetostatic spin-wave modes of a heterogeneous ferromagnetic double layer. *Journal of Applied Physics* **52**, 6824–6829 (1981).

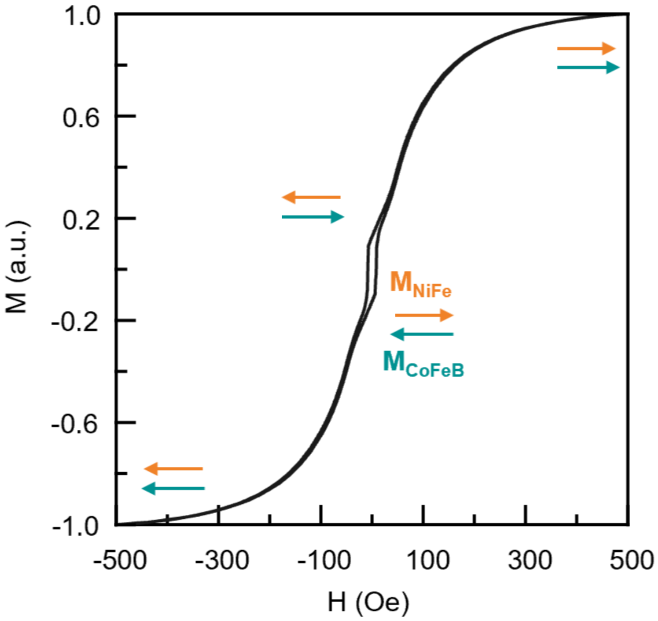

Supplementary Figure 1 | Hysteresis loop of the SAF. Normalized in-plane component of the magnetization of the SAF multilayer employed in the experiment, measured via Vibrating Sample Magnetometry. The orange and teal arrows show the direction of the magnetization in the NiFe and CoFeB layers, respectively.

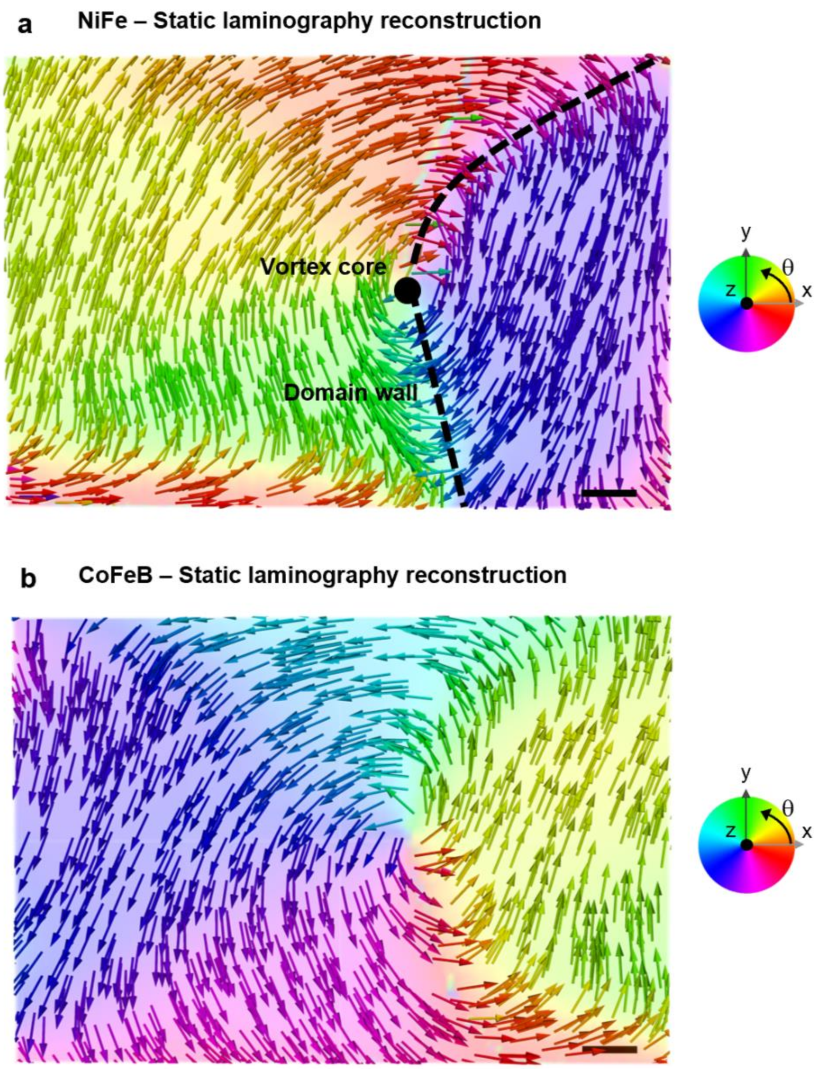

Supplementary Figure 2 | Reconstruction of the static magnetization for NiFe (a) and CoFeB (b), top view. The arrows indicate point-by-point the in-plane magnetization direction. The color-code represents the direction of the in-plane magnetization with respect to *x*. The magnetization in the two layers is antiparallel point-by-point. The curl in the magnetization due to the shape anisotropy, as well as different nanoscale spin textures, indicated by black lines, are visible. Scale bars, 200 nm.

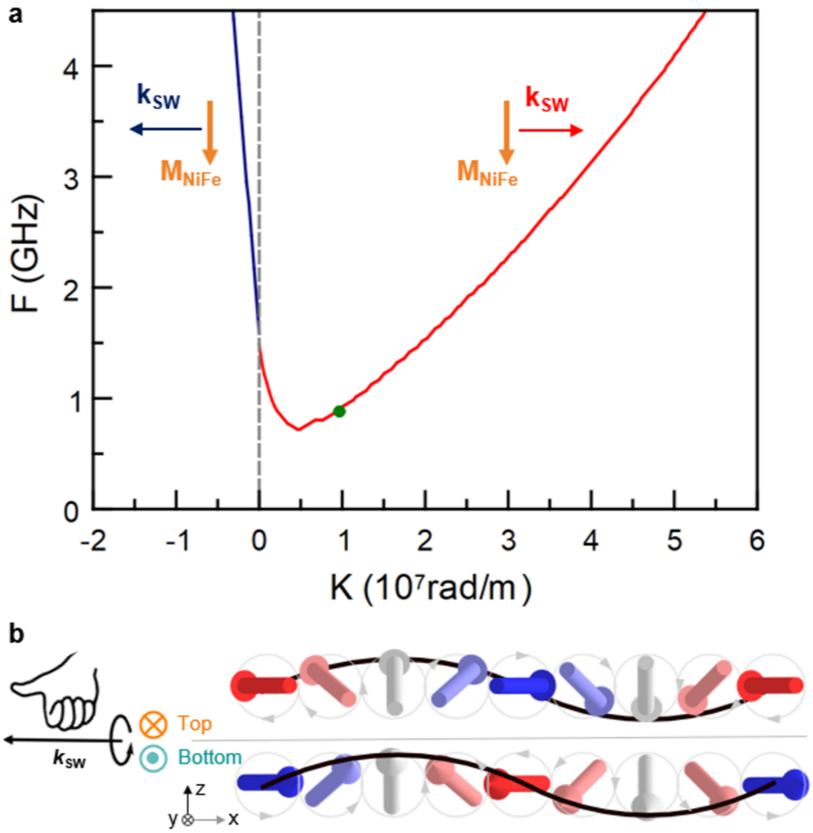

Supplementary Figure 3 | Micromagnetic simulation of the acoustic SW mode dispersion in the SAF. a, The magnetization orientation in the top NiFe layer and the propagation direction in both the positive (+*k*, red) and negative (-*k*, blue) branches of the dispersion is highlighted. The green point represents the experimental data point. **b,** Right-hand rule for ($\mathbf{M}_{top}$, $\mathbf{M}_{bot}$, $\mathbf{k}_{sw}$) showing the direction of propagation for the $k > 0$ branch of the acoustic modes.

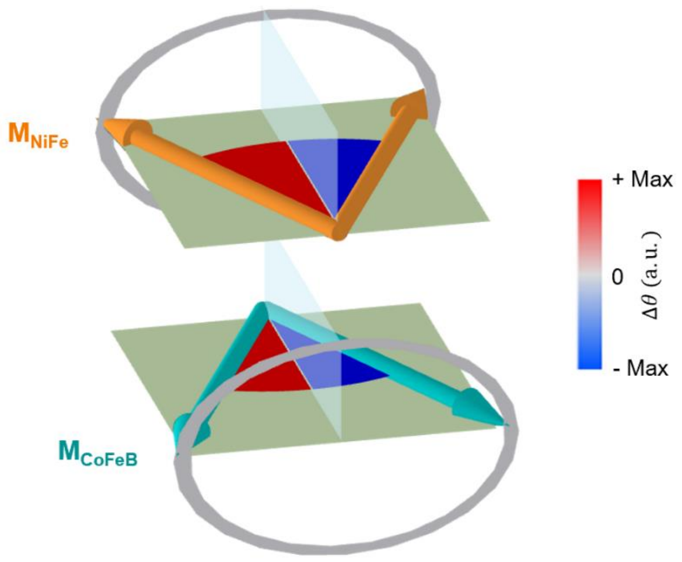

Supplementary Figure 4 | Color-code convention for the dynamic in-plane magnetization. The dynamic magnetization is represented as an orange (teal) arrow for NiFe (CoFeB). The red-blue contrast represents the value of the in-plane dynamic angle $\Delta\theta$, as defined in the main text. To visualize the antiparallel alignment of the in-plane component of the SAF spin-wave modes, the sign convention of $\Delta\theta$ is opposite in the two layers.

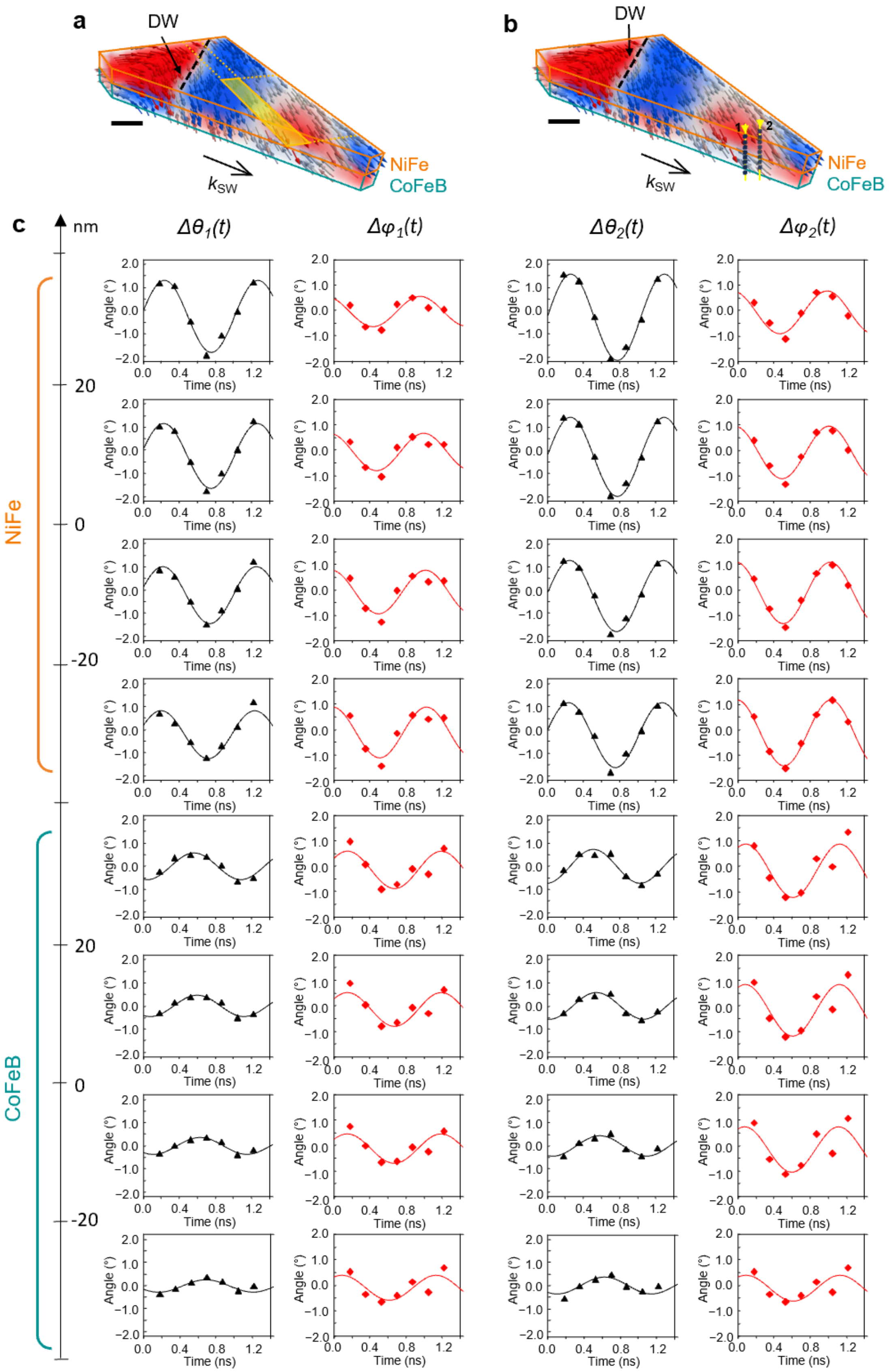

a
DW
NiFe
CoFeB
k_{SW}
b
DW
NiFe
CoFeB
k_{SW}
c
nm
$\Delta\theta_1(t)$
$\Delta\varphi_1(t)$
$\Delta\theta_2(t)$
$\Delta\varphi_2(t)$
NiFe
CoFeB
20
0
-20
Angle (°)
Time (ns)

Supplementary Figure 5 | *z*-dependent spin-wave amplitude profiles. **a**, Snapshot of the time-resolved three-dimensional reconstruction of the region of the microstructure in which the *z*-dependent spin-wave amplitude profiles reported in Figure 3d,e of the main manuscript have been extracted. The amplitude values of each voxel in the yellow rectangular horizontal 480 nm x 120 nm section located at depth *z* were averaged. Scale bar, 150 nm. **b**, Same figure of panel a, highlighting the lines along which the time-traces of 8 points at different depth were analyzed. Scale bar, 150 nm. **c**, Time-traces of the in-plane $\Delta\theta$ (black symbols) and out-of-plane $\Delta\varphi$ (red symbols) dynamics in NiFe and CoFeB, during one period of oscillation for the two different lines of the horizontal sector identified in (**b**).

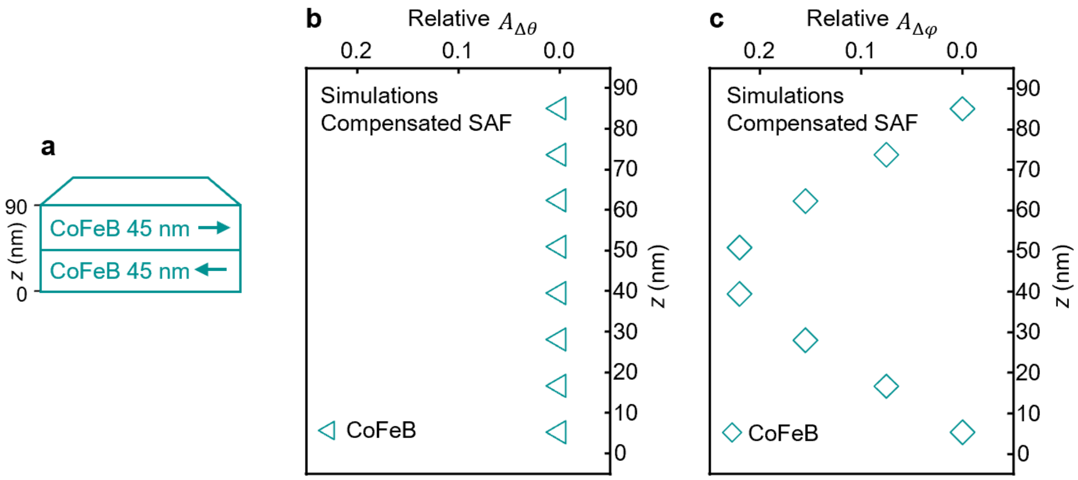

Supplementary Figure 6 | Simulations of SW amplitude and localization through the film thickness in a compensated SAF. a, Sketch of the simulated compensated SAF structure comprising two antiferromagnetically coupled 45 nm thick CoFeB layers. **b**,**c**, Relative variations of the in-plane SW amplitude $A_{\Delta\theta}$ (**b**) and out-of-plane SW amplitude $A_{\Delta\varphi}$ (**c**) for the simulated compensated SAF, as a function of the *z*-position through the thickness of the sample. The simulated SW frequency was $f = 0.86$ GHz.

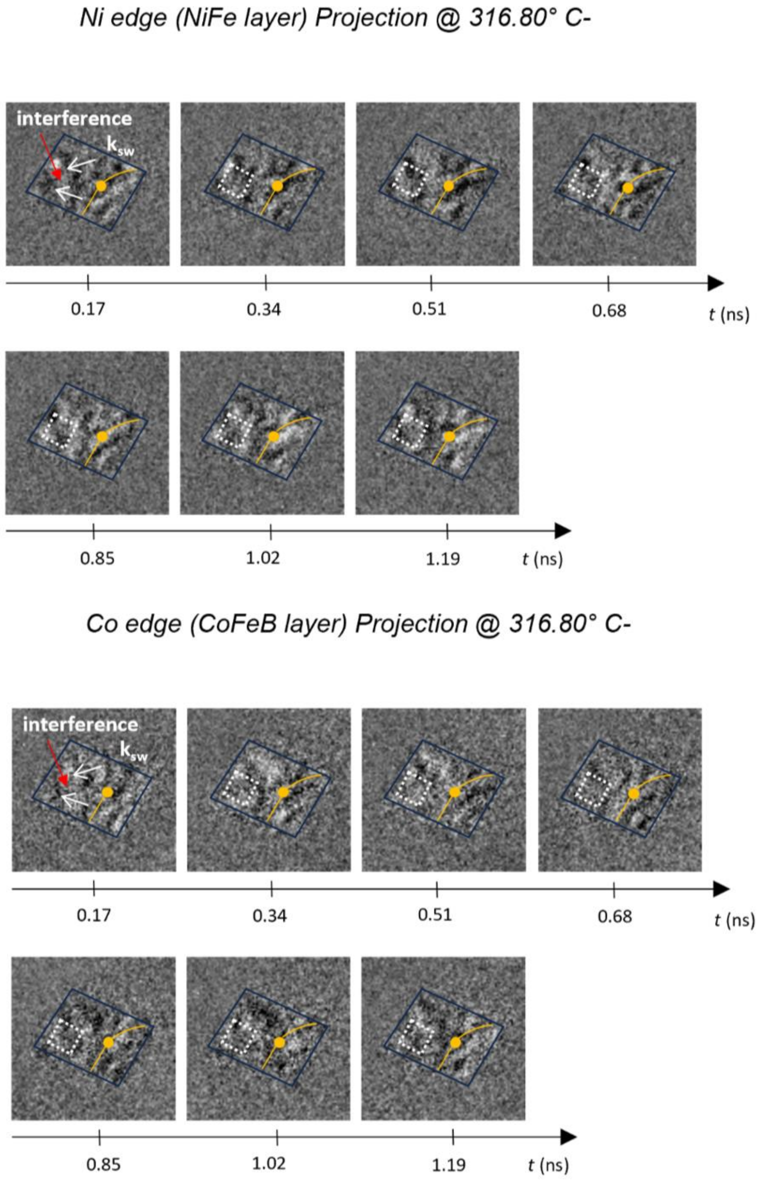

Supplementary Figure 7 | Examples of time-resolved laminography projections. Time-resolved STXM frames acquired for both NiFe and CoFeB at a projection angle of 316.8° and negative circular polarization, showing the interference region of Fig. 4 in the main text. In the top (bottom) panel, the dynamics of the Ni (CoFeB) layer is visualized. As expected, the magnetic contrast associated to spin-wave propagation is reduced in the destructive interference region both in NiFe and CoFeB (indicated by the dashed box).

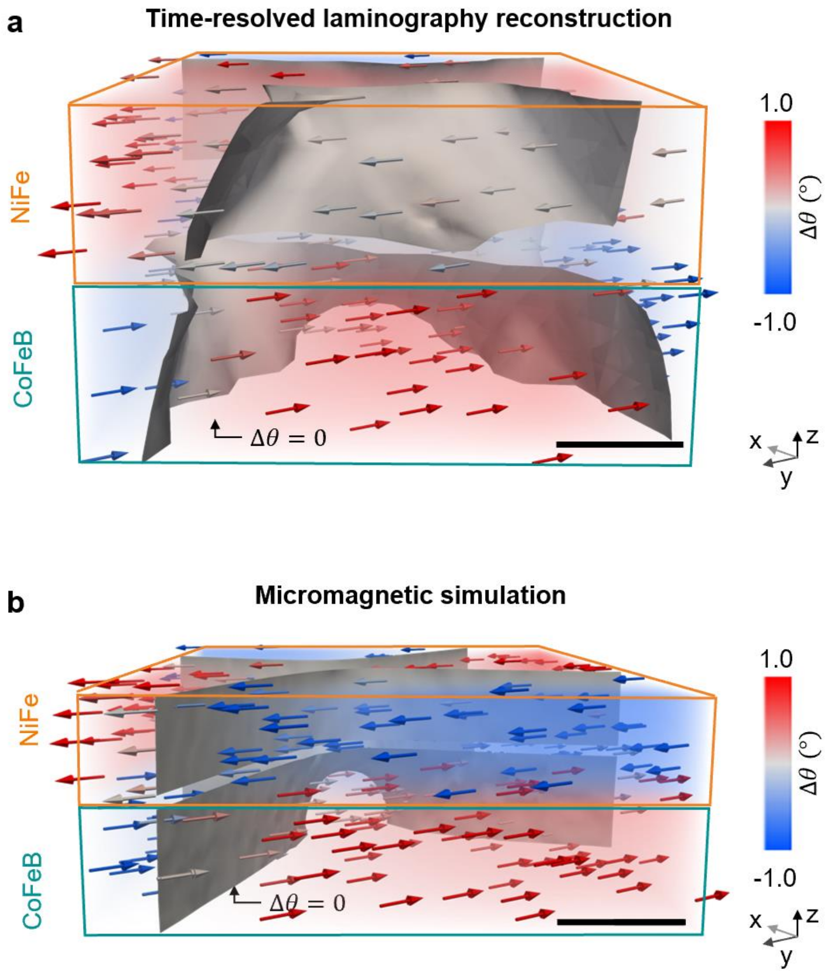

Supplementary Figure 8 | Three-dimensional reconstruction of the interference region (rotated by 90°). **a**, Time-resolved three-dimensional reconstruction of the interference region shown in Figure 4c of the main text, rotated by 90°. **b**, Corresponding micromagnetic simulations of the same region. The color-code indicates the in-plane dynamic angle $\Delta\theta$ both in the experiments and simulations. The arrows represent the dynamic magnetization. Scale bars, 50 nm.

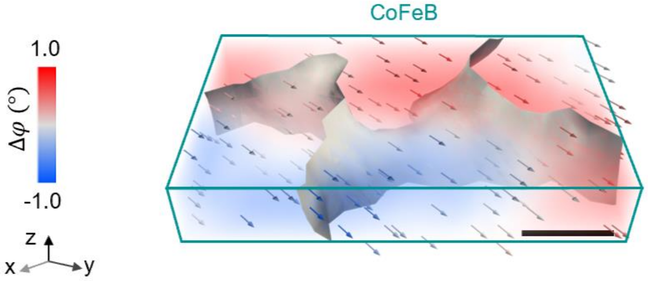

Supplementary Figure 9 | Three-dimensional reconstruction of the interference region in the CoFeB layer, showing the out-of-plane dynamics. Experimental reconstruction of the interference region in the CoFeB layer shown in the main text. The grey surface indicates the saddle-shaped $\Delta\theta = 0$ region. The color code indicates the out-of-plane dynamic angle $\Delta\varphi$. In correspondence of the saddle-shaped destructive interference region both the in-plane ($\Delta\theta$) and the out-of-plane ($\Delta\varphi$) dynamic magnetization vanish. The arrows represent the dynamic magnetization. Scale bars, 50 nm.

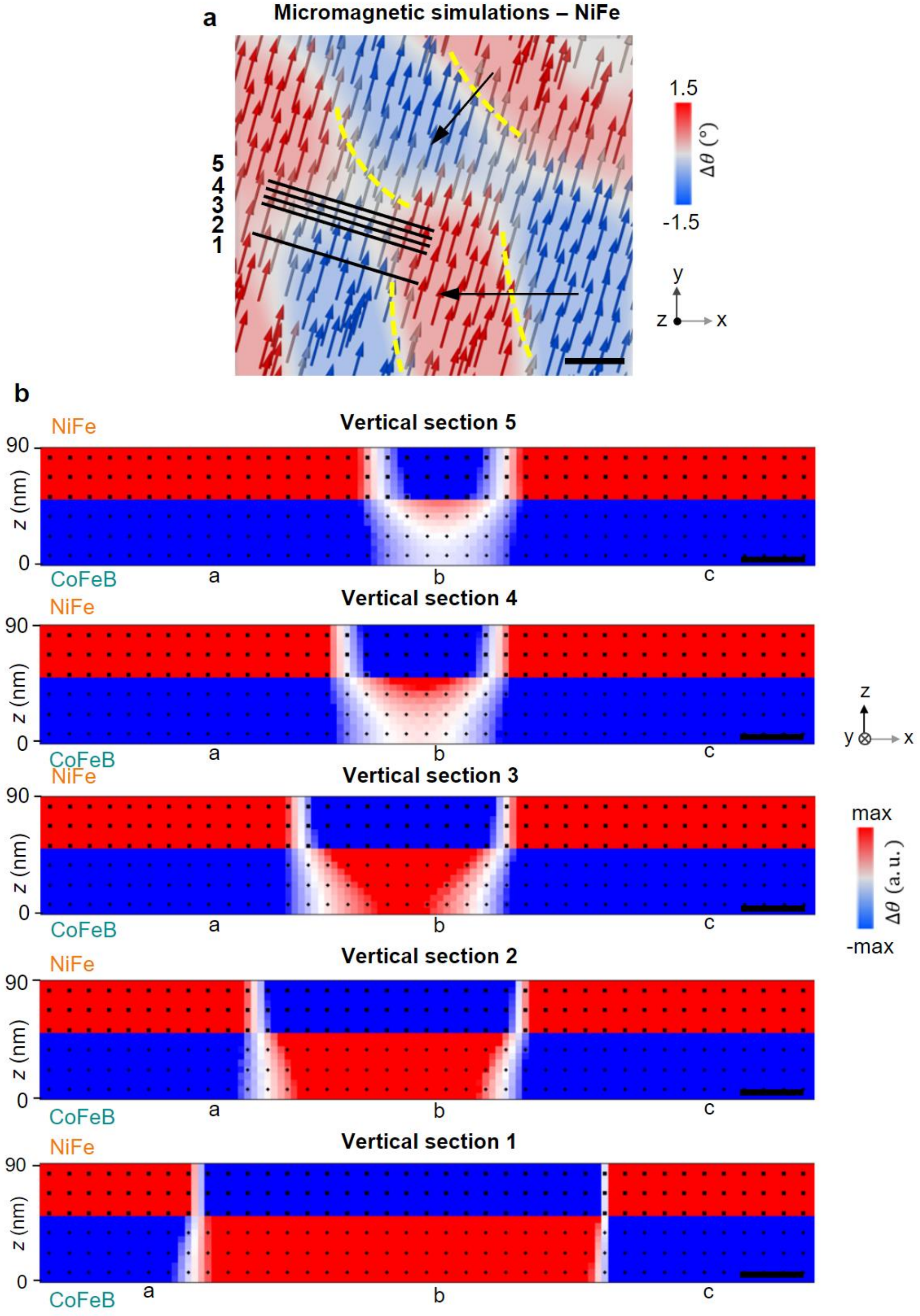

Supplementary Figure 10 | Spatial dependence of the saddle-shaped three-dimensional interference pattern. a, Snapshot of time-resolved micromagnetic simulations of the interference region. Scale bar, 200 nm. **b**, Vertical sections extracted in correspondence of the black numbered lines in panel **a**. The color code indicates the in-plane dynamic angle $\Delta\theta$. Scale bars, 45 nm.

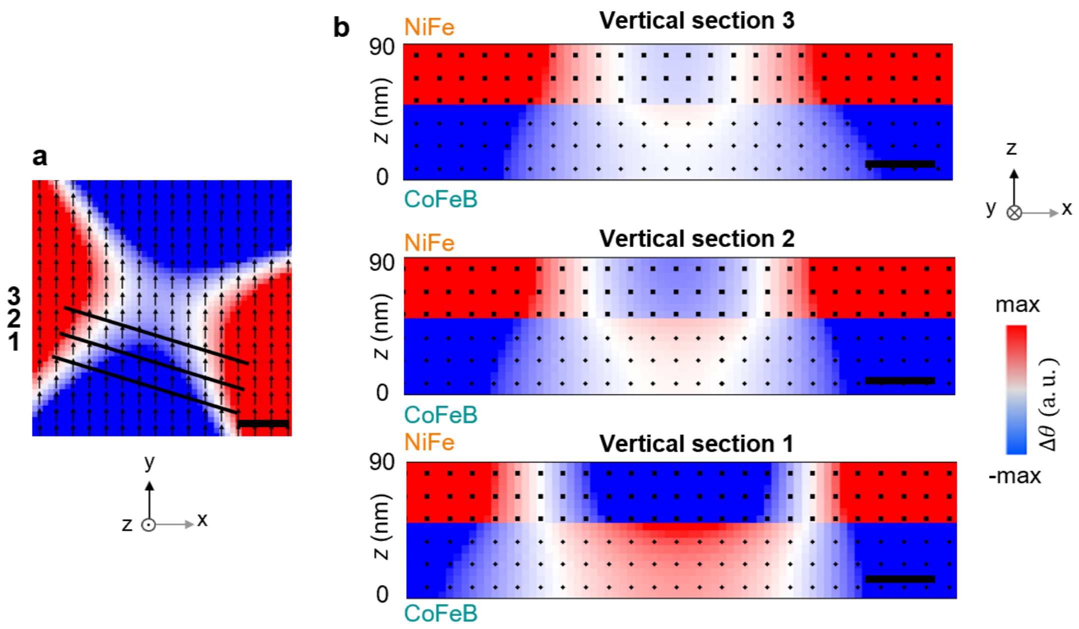

Supplementary Figure 11 | Spatial dependence of the saddle-shaped three-dimensional interference pattern (soft color scale). a, Snapshot of time-resolved micromagnetic simulations of a saddle-shaped interference region. Scale bar, 200 nm. **b**, Vertical sections extracted in correspondence of the black lines in panel **a**. The color code indicates the in-plane dynamic angle $\Delta\theta$. Scale bars, 45 nm.

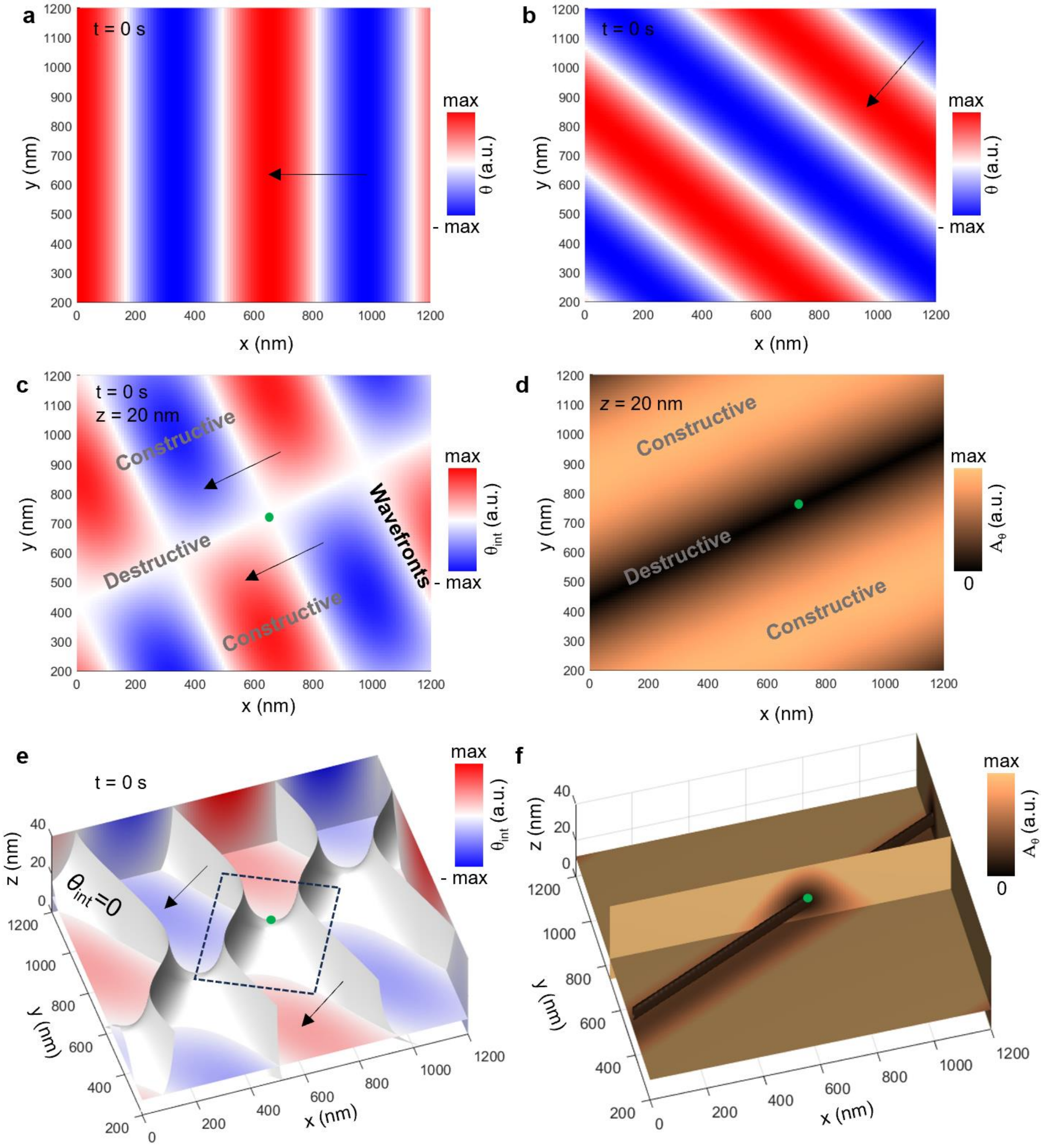

Supplementary Figure 12 | Spatial and temporal evolution of three-dimensional spin-wave interference. **a**, **b**, Top view (*xy* plane) snapshot at $t = 0$ of planar wavefronts of the two individual waves. **a**, Wave 1 $\theta_1(t, \mathbf{r})$, propagating along -*x*. **b**, Wave 2 $\theta_2(t, \mathbf{r})$ propagating from right to left at an angle of 50° with respect to each other. The propagation direction is indicated by the black arrows. **c**, Top view (xy plane) snapshot at $t = 0$ s of the interfering waves $\theta_{\text{int}}(t, \mathbf{r}) = \theta_1(t, \mathbf{r}) + \theta_2(t, \mathbf{r})$. Regions of constructive and destructive interference are indicated. The arrows mark the propagation direction of the wavefronts of the interference pattern. $\theta_{\text{int}} = 0$ regions are in white. **d**, corresponding amplitude $A_{\theta_{\text{int}}}(\mathbf{r})$ map, where destructive (constructive) interference gives rise to amplitude minima (maxima). **e**, *z*-dependence of the interference patterns, featuring the buried saddle-shaped $\theta_{\text{int}} = 0$ surface in correspondence of the green point. As a function of time, the saddle and the wavefronts propagate in the direction of the arrows. The dashed rectangle marks a single saddle, for comparison with Fig. 4c. **f,** Corresponding *z*-dependent $A_{\theta_{\text{int}}}(\mathbf{r})$ amplitude profile, displaying a "buried" destructive interference region (black "tube") located in the middle of the layer, characterized by $A_{\theta_{\text{int}}} = 0$.
