# Supplementary material for: Three-dimensional spin-wave dynamics, localization and interference in a synthetic antiferromagnet
Source: Nat Commun. 2024 Apr 9;15:3057. doi: 10.1038/s41467-024-47339-9 (PMC11004151; doi:10.1038/s41467-024-47339-9)
Supplement: Supplementary file 2 — Description of Additional Supplementary Files [file 41467_2024_47339_MOESM2_ESM.pdf]

Supplementary Movie 1
Time-resolved experimental precession of the magnetization in NiFe. Time-resolved experimental precession of the magnetization extracted from the black dashed rectangle of Fig. 2b in NiFe, associated to the spin-wave propagation. The red-blue color-code represents the in-plane dynamic angle $\Delta\theta$, as defined in the main text; the arrows with the black contour represent the static magnetization. The video consists of 7 frames (0.17 ns/frame) repeated 4 times for better visualization of the precession. Scale bar, 50 nm.

Supplementary Movie 2
Time-resolved experimental precession of the magnetization in NiFe enhanced by a factor of 5. Time-resolved experimental precession of the magnetization extracted from the from the black dashed rectangle of Fig. 2b in NiFe (first frame reported in Fig. 2a), associated to the spin-wave propagation. The in-plane and out-of-plane dynamics are enhanced by a factor of 5 for better visualization. The red-blue color-code represents the in-plane dynamic angle $\Delta\theta$, as defined in the main text; the arrows with the black contour represent the static magnetization. The video consists of 7 frames (0.17 ns/frame) repeated 4 times for better visualization of the precession. Scale bar, 50 nm.

Supplementary Movie 3
Time-resolved experimental precession of the magnetization in CoFeB. Time-resolved experimental precession of the magnetization extracted from the black dashed rectangle of Fig. 2b in CoFeB, associated to the spin-wave propagation. The red-blue color-code represents the in-plane dynamic angle $\Delta\theta$, as defined in the main text; the arrows with the black contour represent the static magnetization. The video consists of 7 frames (0.17 ns/frame) repeated 4 times for better visualization of the precession. Scale bar, 50 nm.

Supplementary Movie 4
Time-resolved experimental precession of the magnetization in CoFeB enhanced by a factor of 5. Time-resolved experimental precession of the magnetization extracted from the black dashed rectangle of Fig. 2b in CoFeB (first frame reported in Fig. 2c), associated to the spin-wave propagation. The in-plane and out-of-plane dynamics are enhanced by a factor of 5 for better visualization. The red-blue color-code represents the in-plane dynamic angle $\Delta\theta$, as defined in the main text; the arrows with the black contour represent the static magnetization. The video consists of 7 frames (0.17 ns/frame) repeated 4 times for better visualization of the precession. Scale bar, 50 nm.

Supplementary Movie 5
Top-view of time-resolved experimental spin-wave propagation in the NiFe layer. Time-resolved experimental magnetization associated to the spin-wave propagation in the NiFe layer. The arrows represent point-by-point the dynamic magnetization, mediated over the layer thickness. The red-blue color-code represents the value of the in-plane dynamic angle AO, as defined in the main text. The video consists of 7 frames (0.17 ns/frame) repeated 4 times for better visualization of the dynamics. Scale bar, 175 nm.

Supplementary Movie 6
Top-view of time-resolved experimental spin-wave propagation in the CoFeB layer. Time-resolved experimental magnetization associated to the spin-wave propagation in the CoFeB layer. The arrows represent point-by-point the dynamic magnetization, mediated over the layer thickness. The red-blue color-code represents the value of the in-plane dynamic angle AO, as defined in the main text. The video consists of 7 frames (0.17 ns/frame) repeated 4 times for better visualization of the dynamics. Scale bar, 175 nm.

Supplementary Movie 7
Time-resolved 3D reconstruction of spin-wave propagation in the Synthetic Antiferromagnet. Time-resolved experimental 3D reconstruction of the magnetization associated to the propagating spin waves for both NiFe and CoFeB layers, extracted from the triangular area of Extended Data Figure 5 (first 4 frames reported in Fig. 3a). The arrows represent point-by-point the dynamic magnetization. The red-blue color-code represents the value of the in-plane dynamic angle AO, as defined in the main text. The video consists of 7 frames (0.17 ns/frame) repeated 4 times for better visualization of the dynamics. Scale bar, 150 nm.

Supplementary Movie 8
Time-resolved 3D reconstruction of spin-wave propagation in the Synthetic Antiferromagnet enhanced by a factor of 5. Time-resolved experimental 3D reconstruction of the magnetization associated to the propagating spin waves for both NiFe and CoFeB layers, extracted from the triangular area from Extended Data Figure 5. The arrows represent point-by-point the dynamic magnetization. The red-blue color-code represents the value of the in-plane dynamic angle AO, as defined in the main text. The spin precession is amplified by 5 times for better visualization. The video consists of 7 frames (0.17 ns/frame) repeated 4 times for better visualization of the dynamics. Scale bar, 150 nm.

Supplementary Movie 9
Full revolution of the experimental time-resolved 3D reconstruction of the generated spin-wave interference in the Synthetic Antiferromagnet. 360° revolution of the experimental time-resolved 3D reconstruction of the SW interference in NiFe and CoFeB (front view reported in Fig. 4c). The red-blue color-code represents the in-plane dynamic angle AO; the arrows show the dynamic magnetization; the grey surface corresponds to AO = 0°. Scale bar, 25 nm.

Supplementary Movie 10

Full revolution of the time-resolved 3D micromagnetic simulation of the generated spin-wave interference in the Synthetic Antiferromagnet. 360° revolution of the time-resolved 3D micromagnetic simulation of the SW interference in NiFe and CoFeB (front view reported in Fig. 4e). The red-blue color-code represents the in-plane dynamic angle A*O*; the arrows show the dynamic magnetization; the grey surface corresponds to A*O* = 0°. Scale bar, 25 nm.
